# Phenylalanine and Tyrosine as Exogenous Precursors of Wheat (*Triticum aestivum* L.) Secondary Metabolism through PAL-Associated Pathways

**DOI:** 10.3390/plants9040476

**Published:** 2020-04-09

**Authors:** Pavel Feduraev, Liubov Skrypnik, Anastasiia Riabova, Artem Pungin, Elina Tokupova, Pavel Maslennikov, Galina Chupakhina

**Affiliations:** Institute of Living Systems, Immanuel Kant Baltic Federal University, 236000 Kaliningrad, Russia; lskrypnik@kantiana.ru (L.S.); avryabova@stud.kantiana.ru (A.R.); apungin@kantiana.ru (A.P.); ishtrants@kantiana.ru (E.T.); pmaslennikov@kantiana.ru (P.M.); gchupakhina@kantiana.ru (G.C.)

**Keywords:** *Triticum aestivum* L., phenolic compounds, lignin, phenylalanine, tyrosine, PAL, TAL

## Abstract

Reacting to environmental exposure, most higher plants activate secondary metabolic pathways, such as the metabolism of phenylpropanoids. This pathway results in the formation of lignin, one of the most important polymers of the plant cell, as well as a wide range of phenolic secondary metabolites. Aromatic amino acids, such as phenylalanine and tyrosine, largely stimulate this process, determining two ways of lignification in plant tissues, varying in their efficiency. The current study analyzed the effect of phenylalanine and tyrosine, involved in plant metabolism through the phenylalanine ammonia-lyase (PAL) pathway, on the synthesis and accumulation of phenolic compounds, as well as lignin by means of the expression of a number of genes responsible for its biosynthesis, based on the example of common wheat (*Triticum aestivum* L.).

## 1. Introduction

Wheat (*Triticum aestivum* L.) is one of the essential agricultural plants worldwide; it is a leading crop both in terms of land used for its cultivation and the level of its global consumption. Most of the crops, including wheat, are grown as monocultures. This method of growing involves increased stress on plants from both biotic and abiotic factors [1,2,3]. Studying plant metabolism characteristics is a natural step towards the improvement of growth and productivity of plants, especially through the intensification of secondary metabolic processes, such as synthesis of phenolic compounds and lignin—an important element of cell walls.

Phenolic compounds are one of the main classes of secondary metabolites in plant cells. They are involved in a number of protective and regulatory processes in plants, ensuring their durability, steady growth, and tissue development [4,5].

Lignin is the main component of plant cell walls [5,6,7]. Lignin synthesis is a key innovation in the evolution of vascular plants, in which this phenolic polymer plays a significant role in providing structural support, facilitating water transport, and creating physical barriers, thereby acting as a non-specific factor of plant immunity, which is of fundamental importance in the early stages of plant ontogenesis [8,9].

For the synthesis of numerous secondary metabolites, including monolignols, as building blocks for lignin structures, and flavonoids, the phenylpropanoid pathway is important because it provides them with the “material” [10]. There are three enzymes in the phenylpropanoid pathway: phenylalanine ammonia-lyase (PAL), cinnamate-4-hydroxylase (C4H), and 4-coumarate—CoA ligase (4CL), which catalyze the first three steps in the reaction sequence of the phenylpropanoid pathway. It has been shown that genetic inhibition of *PAL*, *C4H*, and *4CL* genes significantly reduces the phenolic compounds content in the studied plant species [11,12,13,14]. For the final step of lignin biosynthesis, peroxidases and laccases play a crucial role. They are assumed to be key enzymes that catalyze monolignol polymerization, although experimental data are incomplete. Studies of genes encoding peroxidase and laccase in Arabidopsis have demonstrated a close relationship between these enzymes and lignin accumulation in secondary cell walls [15].

Among the enzymes mentioned above, the level of phenylalanine ammonia-lyase (PAL) is particularly significant. PAL is the first regulatory enzyme in the metabolism of phenylpropanoids [16]. In fact, it enables the “switching” from the primary to the secondary metabolism of the plant and leads to the formation of a wide range of secondary metabolites based on a phenylpropanoid skeleton [11]. PAL can be activated under various environmental impacts, such as mechanical damage to plant tissues, increased concentrations of heavy metals in the soil, and deficiency of main components in plant nutrition [17]. This makes it an excellent inductor that triggers reactions aimed at increasing plant resistance. This enzyme found in cereals has a special feature of being able to include tyrosine into metabolic processes along with phenylalanine [4,5,18]. A recent study conducted on *Brachypodium distachyon* cereal model has shown that phenylpropanoid biosynthesis can proceed through fewer stages. In this process, phenolics are produced from tyrosine, which is directly converted into p-coumarate by bifunctional phenylalanine and tyrosine ammonia-lyase (PTAL). Thus, the shortened phenylpropanoid pathway does not include the C4H-catalyzed step [19].

Consequently, it is safe to assume that both L-phenylalanine and L-tyrosine can play a role of specific substrates in PAL/PTAL-catalyzed reactions in wheat plants and intensify the formation of secondary metabolites, such as phenols and lignin, which can be used as a tool to increase the production of natural plant products.

Considering the above, the objective of this study was to get a better understanding of the effects of phenylalanine and tyrosine, involved in plant metabolism through the PAL pathway, on the synthesis and accumulation of phenolic compounds, as well as lignin by means of the expression of a number of genes responsible for its biosynthesis in wheat plants (*Triticum aestivum* L.).

## 2. Results 

### 2.1. Free Phenylalanine and Tyrosine Content in Wheat 

Initially, it was necessary to assess the income level of targeted amino acids (phenylalanine and tyrosine) in experimental plants and to identify the optimal concentration of precursors to adjust their level in nutrient media at subsequent stages of the experiment. The second stage of the study included the assessment of enzymatic activity and the level of gene expression directly or indirectly associated with the involvement of amino acids in secondary metabolism, in particular the synthesis of monolignol precursors and bioflavonoids. This stage involved the use of the optimal concentration of amino acids in a nutrient solution with fixed exposure time.

**Figure 1:**
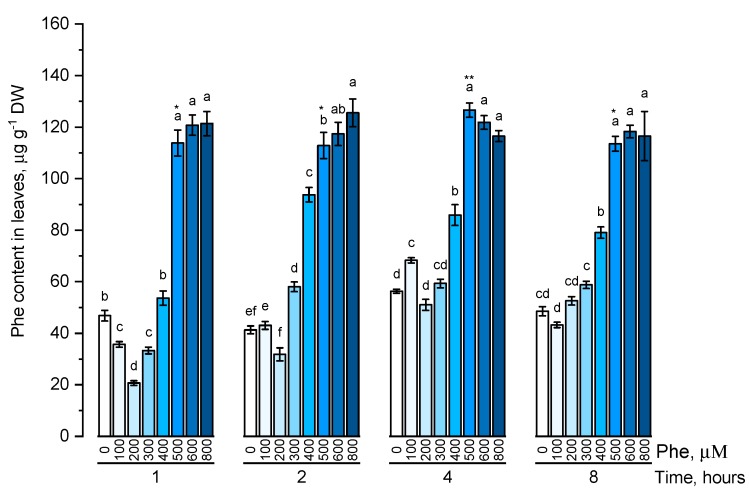
Intracellular content of free phenylalanine in wheat samples incubated on phenylalanine-enriched medium containing various concentrations of the amino acid (100, 200, 300, 400, 500, 600, and 800 μM) for 1, 2, 4, 8 h. Bars marked with different letters show significant differences for the defined time of cultivation with phenylalanine; asterisk * indicates significant differences among plants exposed for a different time at 500 μM (*p* ≤ 0.05, Tukey’s test).

**Figure 2:**
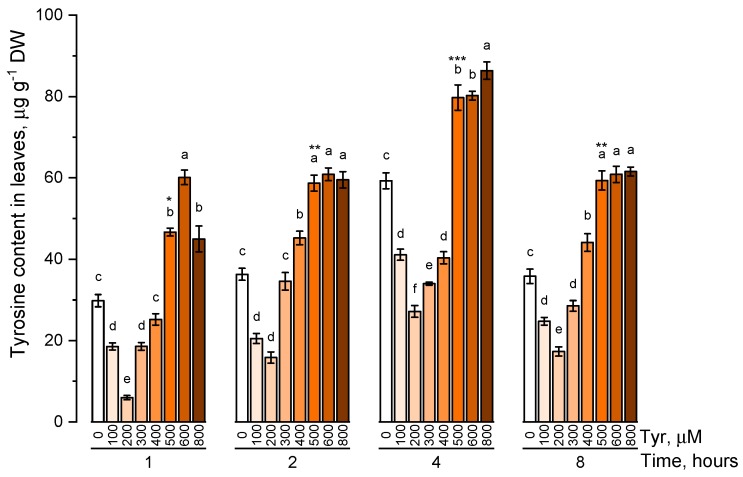
Intracellular content of free tyrosine in common wheat incubated on tyrosine-enriched medium containing various concentrations of the amino acid (100, 200, 300, 400, 500, 600, and 800 μM) for 1, 2, 4, 8 h. Bars marked with different letters show significant differences for the defined time of cultivation with tyrosine; asterisk * indicates significant differences among plants exposed for a different time at 500 μM (*p* ≤ 0.05, Tukey’s test).

The endogenous level of phenylalanine (Figure 1) and tyrosine (Figure 2) was estimated in plants that were cultivated on nutrient media containing 100, 200, 300, 400, 500, 600, 800 μM of the corresponding amino acids at different exposure times (1, 2, 4, and 8 h).

The dynamics of phenylalanine accumulation was mostly linear. The level of free phenylalanine, although being significantly different depending on the exposure time, did not fluctuate significantly in almost all concentration options. The content of intracellular phenylalanine did not significantly increase when the maximum concentrations (600 μM and 800 μM) of this amino acid in the solution were used.

Under conditions of low tyrosine concentrations in the nutrient solution (100, 200 μM), its endogenous concentration significantly (*p* ≤ 0.05) decreased in comparison with the control. An increase in the concentration of tyrosine in solution (300 μM or more) led to a gradual increase in the concentration of tyrosine in plant tissues. The internal level of the studied amino acid reached a peculiar plateau at tyrosine concentrations in solution of 500 μM or higher. Such a pattern in the accumulation of tyrosine was observed regardless of the exposure time of plants to nutrient solutions. However, the maximum pool of intracellular free tyrosine, regardless of the concentration of this amino acid in the nutrient medium, was observed in a 4 h exposure. The concentration of 500 μM turned out to be the optimal concentration of the tyrosine-supplemented medium.

The endogenous level of amino acids did not change significantly or did not change at all, with an increase in a concentration above 500 μM and with an increase in exposure time on a nutrient medium for more than 4 h.

### 2.2. Phenolic Compound Content

**Figure 3:**
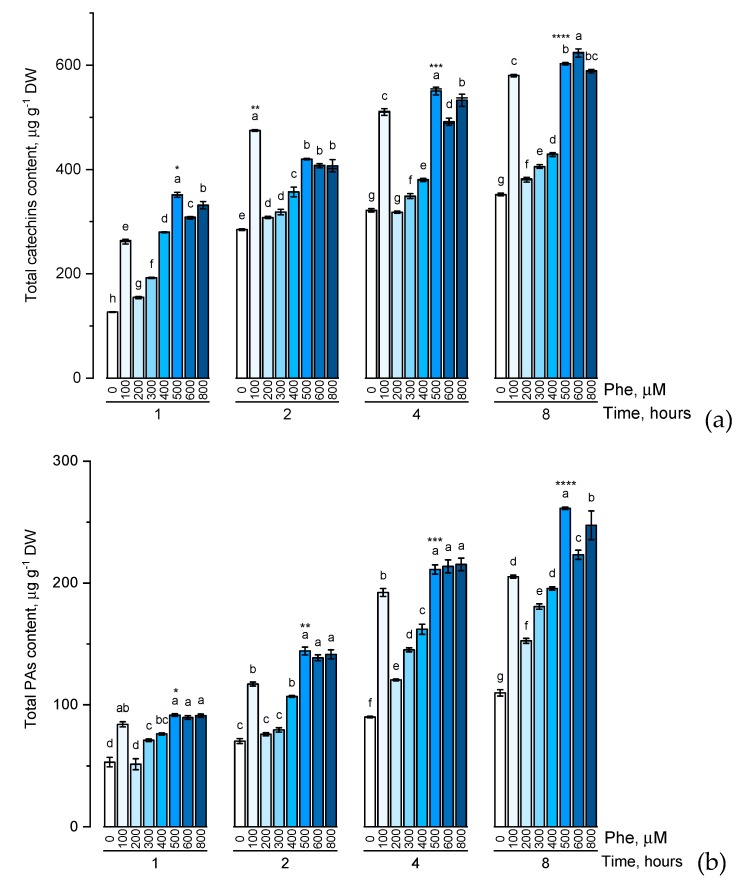

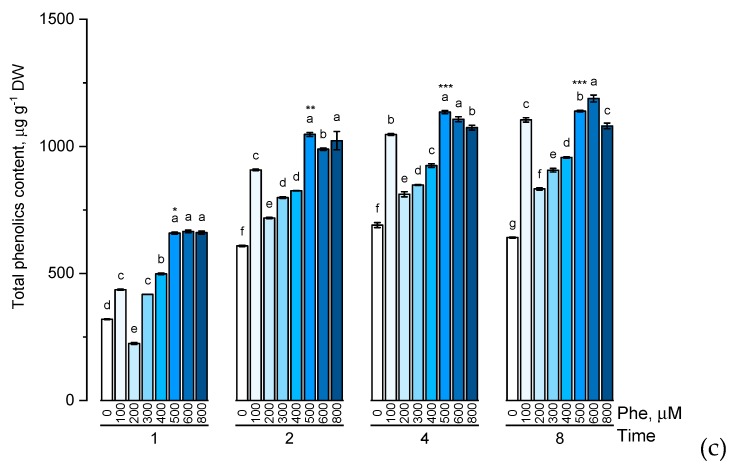
Accumulation of total catechins content (**a**), total proanthocyanidins content (**b**), total phenolic content (**c**) in wheat samples incubated on phenylalanine-enriched medium containing various concentrations of the amino acid (100, 200, 300, 400, 500, 600, and 800 μM) for 1, 2, 4, 8 h. Bars marked with different letters show significant differences for the defined time of cultivation with phenylalanine; asterisk * indicates significant differences among plants exposed for a different time at 500 μM (*p* ≤ 0.05, Tukey’s test).

The level of phenolic compounds is an excellent marker of the intake of phenylalanine and tyrosine, which can act as specific precursors in the biosynthesis of a number of simple phenolic components of the cell, as well as rather complex condensed forms of flavonoids. The level of total catechins content, total proanthocyanidins content, and total phenolic content in the presence of various concentrations of phenylalanine was evaluated (Figure 3).

There was a common trend in the accumulation of the studied groups of phenolic compounds in the presence of phenylalanine of various concentrations (100, 200, 300, 400, 500, 600, and 800 μM) characterized by a gradual increase in the level of these biologically active components with an increase of phenylalanine concentration in the solution. However, it is worth noting that the change in the accumulation of flavonoids with an increase in the concentration of phenylalanine in the nutrient medium from 500 μM to 800 μM appeared not to be dramatic. Another common trend in the accumulation of the studied groups of phenolic compounds was reaching the concentration plateau after 4 h exposure of the plants to solutions containing phenylalanine. 

**Figure 4:**
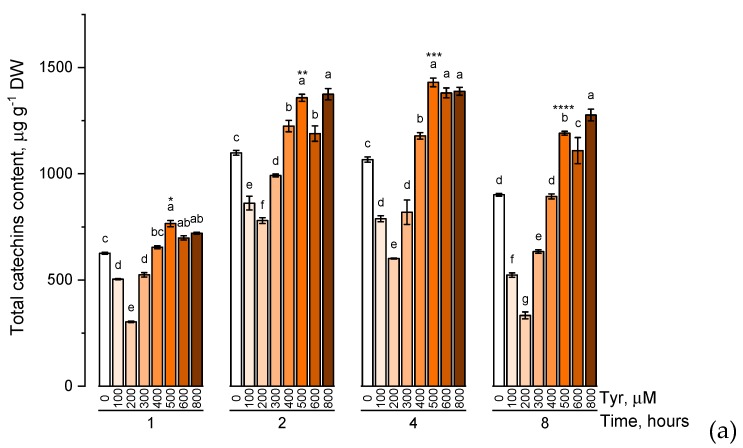

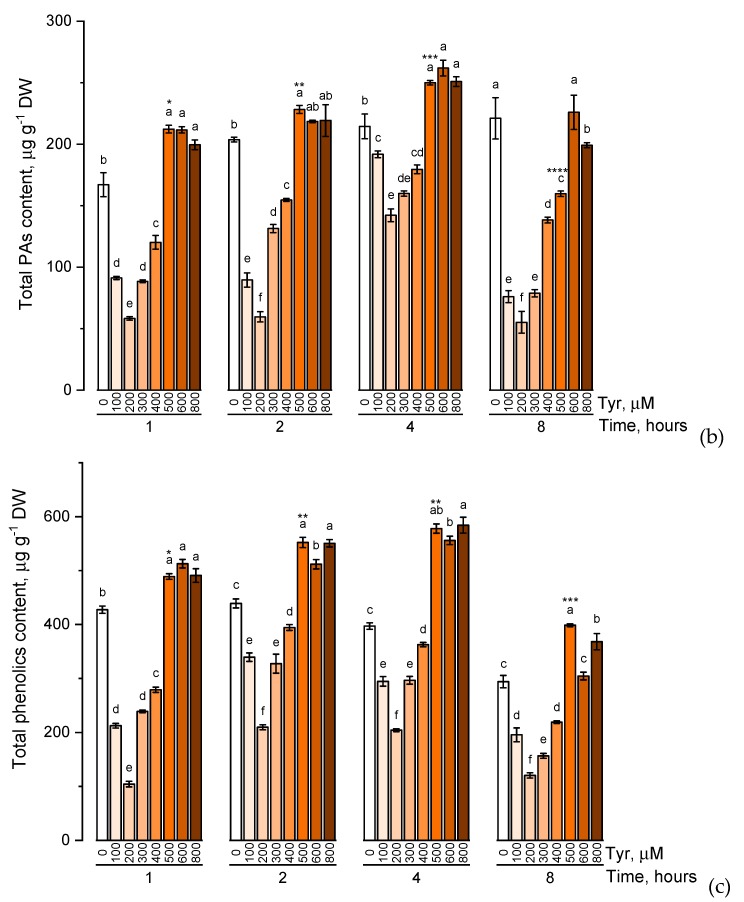
Accumulation of total catechins content (**a**), total proanthocyanidins content (**b**), total phenolic content (**c**) in wheat samples incubated on tyrosine-enriched medium containing various concentrations of the amino acid (100, 200, 300, 400, 500, 600, and 800 μM) for 1, 2, 4, 8 h. Bars marked with different letters show significant differences for the defined time of cultivation with tyrosine; asterisk * indicates significant differences among plants exposed for a different time at 500 μM (*p* ≤ 0.05, Tukey’s test).

The effect of various tyrosine concentrations on the accumulation of phenolic compounds had also been evaluated on experimental wheat plants (Figure 4).

When the experimental plants were exposed to different concentrations of tyrosine (100, 200, 300, 400, 500, 600, and 800 μM), the accumulation profiles of the studied groups of phenolic compounds were similar and characterized by a gradual increase in the level of these biologically active components with an increase in the concentration of tyrosine in the solution (a similar trend was observed in exposure to phenylalanine). However, when the plants were exposed to a tyrosine-supplemented nutrient medium for 8 h, there was a decrease in the concentration of catechins, proanthocyanidins, and total phenols compared with the plants that were exposed to the nutrient medium for 4 h. In addition, as in the case of phenylalanine, an increase in the concentration of tyrosine in the nutrient solution above 500 μM did not lead to a significant increase in the accumulation of these groups of phenolic compounds.

The transfer of experimental plants to nutrient media with the addition of phenylalanine led to a significant increase in the level of the studied phenolic substances already at a concentration of the active substance of 100 μM, and this trend continued throughout the exposure period (for 8 h).

However, when plants were transferred to a medium containing tyrosine, there was no significant increase in the level of phenolic compounds under conditions of low precursor concentration (100, 200 μM).

One of the results of these tests was the identification of certain time and concentration optima of introduced precursors. Thus, the treatment of plants with a 500 μM solution of phenylalanine and tyrosine up to 4 h led to a significant (*p* ≤ 0.05) increase (compared with other experimental variants) of the evaluated compounds.

### 2.3. Enzyme Activity 

In order to reduce the number of experimental points when evaluating the enzymatic activity and gene expression involved in lignin and flavonoid biosynthesis, only those amino acid concentrations and exposure times (500 μM for both amino acids, 4 h) that best affected phenolic compounds accumulation and the total pool of free tyrosine and phenylalanine in the experimental plants were chosen.

**Figure 5:**
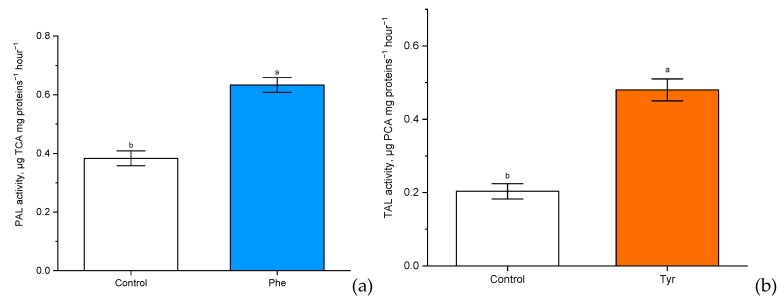
The activity of phenylalanine ammonia-lyase (PAL) (µg trans-cinnamic acid (TCA) mg proteins^−1^ h^−1^) (**a**) and tyrosine ammonia-lyase (TAL) (µg p-coumaric acid (PCA) mg proteins^−1^ h^−1^) (**b**) in wheat samples incubated on the nutrient medium supplemented with 500 μM of phenylalanine and tyrosine respectively, for 4 h. Bars marked with different letters show significant differences at (*p* ≤ 0.05) according to Tukey’s test.

**Figure 6:**
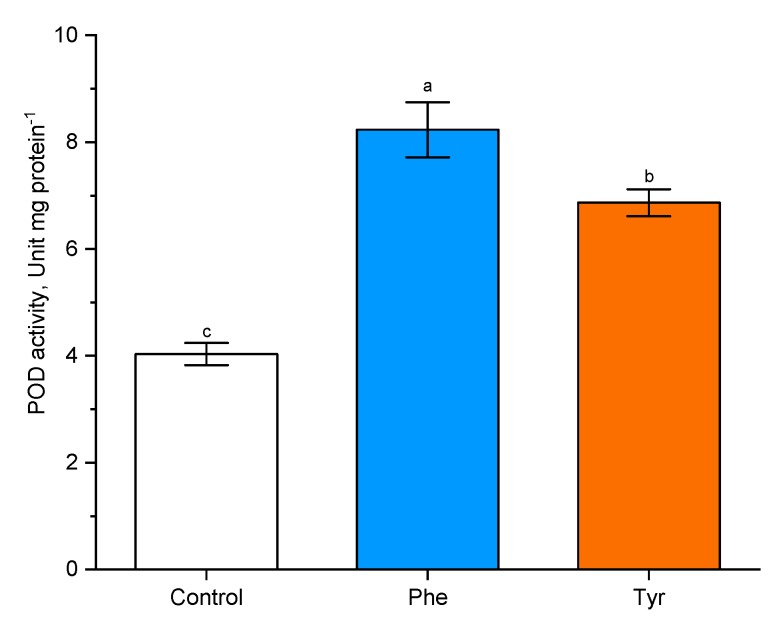
Effects of phenylalanine and tyrosine on POD activity. Bars marked with different letters show significant differences at (*p* ≤ 0.05) according to Tukey’s test.

Enzymes associated with the metabolism of phenylalanine and tyrosine in plants were chosen, either directly as PAL and tyrosine ammonia-lyase (TAL) (Figure 5), catalyzing the conversion of the corresponding substrates, or indirectly as peroxidase (POD) (Figure 6), participating in the crosslinking of monolignol fragments.

There was a significant (*p* ≤ 0.05) increase in PAL and TAL activity compared with the control. Experimental “Vanek” wheat samples, transferred to a nutrient medium containing 500 μM of phenylalanine and tyrosine and exposed under such conditions for 4 h, showed a significantly more than two-fold increase in PAL activity (0.5 µg TCA mg proteins^−1^ h^−1^ in comparison with the level of control plants: 0.2 µg TCA mg proteins^−1^ h^−1^). Phenylalanine ammonia-lyase of cereals also had tyrosine ammonia-lyase activity. It was worth noting that the rate of involvement in tyrosine metabolism through TAL activity (0.65 µg PCA mg proteins^−1^ h^−1^ in comparison with the level of control plants: 0.4 µg PCA mg proteins^−1^ h^−1^) was slightly lower than the rate of involvement in phenylalanine metabolism through PAL.

In order to analyze the effects of phenylalanine and tyrosine (both 500 µM) on POD activity, experimental wheat plants were treated with amino acid solutions for 4 h. It was shown that POD activity increased in the presence of both effectors in the medium.

Plants transferred to the phenylalanine-supplemented medium were characterized by a more than two-fold increase in POD activity compared with the control (8.5 unit/mg protein in comparison with the level of control plants: 4 unit/mg protein). A similar trend was observed when plants were exposed to tyrosine (7 unit/mg protein in comparison with the level of control plants: 4 unit/mg protein). POD activity in wheat plants in the presence of phenylalanine was higher (*p* ≤ 0.05) than POD activity in plants transferred to the tyrosine-supplemented nutrient medium.

The chosen methods made it possible to determine PAL/TAL activity and POD activity in the same extracts. Thus, the common changes in the activity of each of these enzymes were studied. Exposure of plants to a medium enriched in aromatic amino acids led to a significant (*p* ≤ 0.05) increase in activity despite the fact that these enzymatic systems are included in the metabolism of phenylpropanoids at different stages: PAL/TAL—when the corresponding amino acids were introduced into the metabolism of phenolic compounds, and POD—when lignin structures of plants were formed.

### 2.4. Gene Expression 

**Figure 7:**
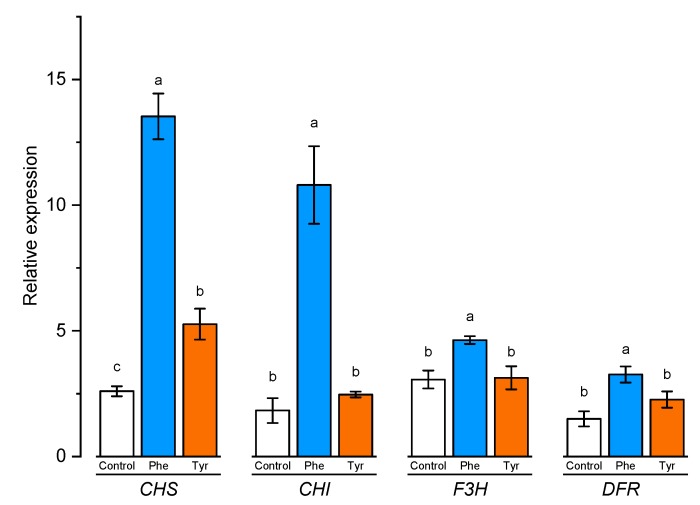
Relative quantification values of CHS, CHI, F3H, and DFR in wheat leaves in the presence of phenylalanine and tyrosine. Bars marked with different letters show significant differences at (*p* ≤ 0.05) according to Tukey’s test. A total of three biological and three technical replicates were conducted.

The level of expression of a number of genes was evaluated in plants exhibited on a nutrient medium supplemented with amino acids, the protein products of which are involved, on the one hand, in lignin synthesis, and, on the other hand, are responsible for the formation of flavonoids. The expression level of chalcone synthase (*CHS*), chalcone isomerase (*CHI*), flavanone 3-hydroxylase (*F3H*), and dihydroflavonol 4-reductase (*DFR*) genes involved in flavonoid biosynthesis was analyzed using qRT-PCR in leaves of wheat samples incubated on the medium containing 500 μM of phenylalanine and tyrosine, respectively, for 4 h (Figure 7).

*CHS* and *CHI* are the first genes that encode the early and unbranched segment of flavonoid biosynthesis. As shown in Figure 7, the relative expression of *CHS* tended to increase the number of transcripts in the presence of amino acids. The results showed that plants transferred to the medium supplemented with phenylalanine and tyrosine had a significantly (*p* ≤ 0.05) higher level of expression of chalcone synthase gene (7 and 2 times, respectively). Chalcone isomerase gene expression level was significantly changed only in plants incubated in medium supplemented with phenylalanine.

*F3R* and *DFR* genes are responsible for the later stage of flavonoid biosynthesis in comparison with *CHS* and *CHI* genes. Rt-PCR results showed that the number of *F3H* transcripts increased slightly in the presence of L-phenylalanine in the nutrient medium. Plants transferred to the phenylalanine-enriched medium had a significantly (*p* ≤ 0.05) higher level of *F3H* gene expression compared with the control (approximately 1.5 times higher than the control group samples). The expression level of the *F3R* and *DFR* genes in the plant incubated on the medium supplemented with tyrosine did not significantly (*p* ≤ 0.05) differ compared to the control. The maximum expression level of the *DFR* gene was recorded in plants transferred to the phenylalanine-supplemented medium (about 2 times compared with the plants from the control group). Thus, the addition of aromatic amino acids to the nutrient medium led to a significant increase in the expression of the *CHS* gene, which encodes a key enzyme of flavonoid biosynthesis.

**Figure 8:**
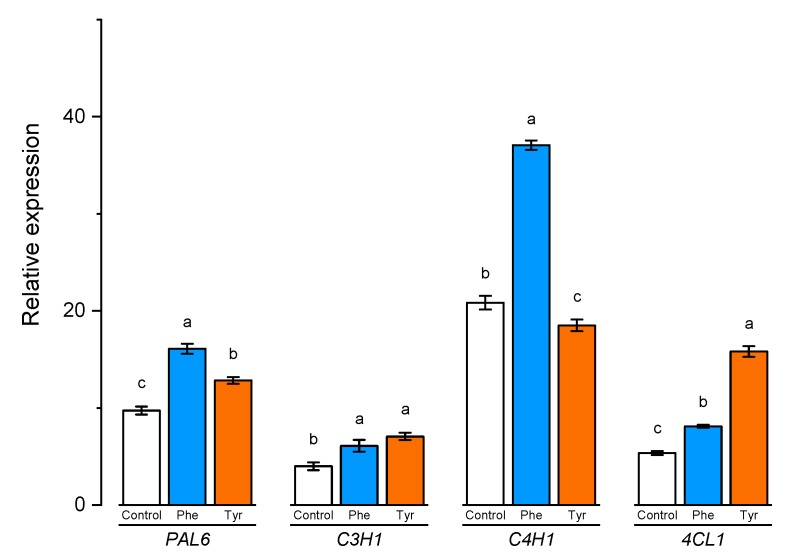
Relative quantification values of *PAL6*, *C4H1*, *4CL1,* and *C3H1* in wheat leaves affected by the phenylalanine and tyrosine levels in the nutrient medium. Bars marked with different letters show significant differences at (*p* ≤ 0.05) according to Tukey’s test.

To better understand the role of aromatic amino acids in the lignification process, the expression of certain lignin biosynthesis genes in wheat samples was analyzed. The expression of *PAL6*, *C4H1*, *4CL1*, and *C3H1* genes correlated significantly with the lignin content in wheat samples (Figure 8).

The expression level of *C3H1* and *4CL1* genes demonstrated a similar trend. The number of transcripts increased slightly in the presence of phenylalanine in the medium. Plants, transferred to solutions containing 500 μM of tyrosine, showed a higher level of transcription of *C3H1* and *4CL1* genes compared with the control (2 and 3 times higher, respectively).

A slight change in the expression level was also observed in the *PAL6* gene when plants were exposed to nutrient media supplemented with phenylalanine and tyrosine for 4 h. Thus, the studied aromatic amino acids led to a 1.5-fold increase in the number of transcripts of this gene, compared with the control.

The number of transcripts of the *C4H1* gene, whose protein product is responsible for the conversion of TCA trans-cinnamic acid and PCA p-coumaric acid, in the cells of plants that were transferred to the phenylalanine-enriched medium turned out to be approximately 2 times higher compared with the control. The expression of the *C4H1* gene in plant cells cultivated in the presence of tyrosine was slightly inhibited.

The presence of both phenylalanine and tyrosine amino acids in the nutrient medium led to a significant change in the transcripts accumulation of the *PAL6* gene. It is worth noting that this gene encodes an enzyme that potentially catalyzes the conversion of both amino acids (phenylalanine and tyrosine), playing a key role in the downstream regulation of phenylpropanoid biosynthesis.

## 3. Discussion

An important feature of aromatic amino acids is their ability to act as primary metabolites, which serve as precursors for many natural (secondary) components of the cell, such as flavonoids, phenolic acids, coumarins, alkaloids, glucosinolates.

Phenylalanine is a substrate for many different secondary metabolites, including phenylpropanoids, flavonoids, anthocyanins, and the cell wall lignin mentioned above. Mutations that inhibit PAL synthesis are usually associated with significant changes in the levels of many phenylpropanoids [20]. 

Tyrosine, in turn, also serves as a precursor for several families of secondary metabolites, including, for example, tocochromanols, plastoquinones, isoquinoline alkaloids, several non-protein amino acids, and probably some phenylpropanoids [21].

The exposure of experimental plants to phenylalanine-enriched medium led to a significant (*p* ≤ 0.05) increase in the level of the phenolic compounds. It was unlikely to be explained by nutritional medium-related stress since the tendency persisted throughout the exposure time (for 8 h). Besides, the exposure of experimental plants to the tyrosine-enriched medium did not lead to any significant changes in the level of the phenolic compounds.

It has been shown that tyrosine feeding induces catechin production at least 2-fold more than phenylalanine. It is known that exogenous Tyr and Phe are supposed to turn into p-coumaric acid on a downward spiral, with the only difference that Phe transformation has two steps (2 reactions), whereas Tyr needs only one [22]. Hypothetically, this time gap could positively affect the levels of catechins as one of the final products of flavonoid synthesis. Multiple organisms using bifunctional PTAL are shown to have an increased affinity between the enzyme and Tyr (based on the Km value) [23]. Unfortunately, there is no data showing the degrees of affinity between PTAL and given amino acids in wheat plants. Higher catechin levels in tyrosine-exposed plants could only be circumstantial evidence. 

Enzymes chosen for this study were in one way or another associated with Phe and Tyr metabolism in plants: directly (PAL and TAL) or indirectly (POD) [24]. It was also shown that PAL:TAL ratio from extracts was about 2:1, while, in experiments conducted on *Brachypodium* [19], this ratio is 4:1. Such differences in the enzyme activities could be explained by species-specificity. For now, there is no data on the degrees of affinity (Km) between PAL and TAL and their target substrates in wheat plants, especially in the “Vanek” type. There are certain observations that indicate tyrosine’s better involvement in metabolism, such as catechin levels in tyrosine-exposed plants. 

The final stage of lignin biosynthesis is the polymerization of monolignols, which occurs with the participation of peroxidases [24]. Peroxidases are shown to be key enzymes that catalyze monolignol polymerization [15]. POD activity significantly increased when plants were exposed to nutrient media supplemented with the studied amino acids. This might be one of the indirect evidence of the activation of lignin synthesis in experimental wheat plants. As of today, there is no clear understanding of how aromatic amino acids, such as Phe and Tyr, can affect gene expression in plants. But there is a general idea that metabolic substrates could influence transcription processes, including gene transcription, resulting in molecular products directly involved in the metabolism of given substrates. It is safe to assume that genes, the expression products of which are involved in the phenylpropanoid pathway, can be regulated by a feedforward mechanism: the increase of encoded enzymes leads to an increase in transcripts level. However, this statement is not so clear and conclusive. Research conducted on Bacillus subtilis has shown that even big changes in flux through the enzymes do not lead to notable changes in enzyme concentrations [25]. Since the expression of various metabolic enzymes is controlled by transcription regulators, which are susceptible to the outside signals (including the chemical signals of possible substrates), it allows us to interpret the majority of expression changes as the metabolic phenotype changes [26].

V-myb myeloblastosis viral oncogene homologs (MYB) are members of the big transcription factors family; they are also the key regulators of the synthesis of phenylpropanoid derivatives, such as monolignols and flavonoids. There is a likely correlation between the level of studied aromatic amino acids and the level of MYB-factors; however, the typical response regulators proving this correlation have not been found yet [27].

It has been shown that the flux through the phenylpropanoid pathway can be regulated by various intermediates of the pathway itself as well. It’s a well-known fact that ammonia lyases can be regulated by intermediates of its own reactions [19,28,29,30]. PAL and TAL functions can be regulated in different ways by various specific intermediates of the lignin biosynthesis pathway [31].

The question arises whether the enzyme encoded by the *PAL6* gene, the expression level of which increases in the presence of both tyrosine and phenylalanine, can be a bifunctional enzyme. Most of the ammonia lyases of aromatic amino acids are homotetramers consisting of four identical subunits. These subunits form one active site containing a modified amino acid cofactor 3,5-dihydro-5-methylidene-4H-imidazol-4-one (MIO). Enzyme-associated MIO is formed by autocatalytic condensation during peptide packing using highly conserved alanine-serine-glycine (ASG) tripeptide motif. MIO is discovered in PAL, TAL, PTAL, histidine ammonia-lyase (HAL), and aminomutases but not in the other members of the L-amino acid-lyase family, such as aspartate-ammonia-lyases [32].

PTAL can use both Tyr and Phe with equal efficiency. It has been noted before that PTAL in monocotyledons has demonstrated PAL/TAL activity ratio between 0.2 and 6 [19,32,33,34].

PTAL’s multi-substrate mechanism is not yet clearly understood. Previous studies have shown that one His-group in PTAL is responsible for the formation of hydrogen bonds with tyrosine’s hydroxyl. As a result of a mutative substitution (His to Phe) in *Rhodobacter sphaeroides*, the enzyme selectivity has switched from TAL to PAL. An opposite substitution (Phe to His) in PAL of Arabidopsis has led to an 18-fold increase of TAL activity and an 80-fold decrease of PAL activity [32]. However, there is evidence suggesting that some other groups, such as the ones enabling the flexibility of the active site lid loops, are necessary for substrate binding. They can also help the PTAL’s catalysis in certain plants [31,35].

Partial alignment of the sequence of PAL and PTAL proteins (BdPTAL1, TaPAL6, TaPAL1) showed that in the key positions (H136F, A263S, V386L, I395L, D433E) determining the selective binding of the substrate, PAL6, selected for analysis, showed significant similarity with *Brachypodium distachyon* PAL1, with a proven catalytic function with respect to two substrates (Figure S1).

In given experimental conditions, in the presence of phenylalanine, the number of transcripts of the *PAL6* gene increased by almost 1.5 times compared with the control; however, the expression level of *PAL6* also increased, albeit slightly, when plants were exposed to a solution containing 500 μM of tyrosine. It is worth noting that previous studies have not revealed tyrosine ammonia-lyase activity for the enzymatic product of this gene. Analysis of all PAL homologs in *Brachypodium distachyon* cereal revealed the presence of one bifunctional enzyme PTAL (BdPTAL1), which additionally displayed TAL activity but had a much higher Km in relation to phenylalanine. It could be assumed that PAL6 *Triticum aestivum* L. also displayed tyrosine ammonia-lyase activity, and the enzyme itself had a lower affinity for this substrate (tyrosine) than for phenylalanine. This reflected the level of expression of this gene in the presence of affecters, and the data on the alignment of amino acid sequences (wheat’s PAL1 metabolizing exclusively phenylalanine was chosen as an additional control point).

Along with the genes of the *PAL* family, a number of genes located downstream play a fundamental role in the biosynthesis of secondary metabolites. The protein product of the *C4H1* gene converts TCA to PCA. When tyrosine is introduced into metabolism through the TAL pathway, this stage is absent since tyrosine is directly converted to PCA [36]. This was confirmed by the expression of the *C4H1* gene whose level significantly increased in the presence of phenylalanine but not tyrosine. The number of transcripts of the *4CL1* gene significantly increased when plants were cultivated on a tyrosine-supplemented nutrient medium. The protein product of the *4CL1* gene enables the conversion of PCA to 4-coumarate – CoA [37]. Since tyrosine was directly converted to PCA in TAL-mediated reactions and directly supplied the substrate for 4-coumarate: CoA ligase, the expression level of 4CL1 was higher in plants exposed for 4 h on tyrosine than in plants cultivated on phenylalanine. It could be assumed that TCA could act as a substrate for a number of substances, and not all of its pool goes to the synthesis of PCA [38]. P-coumarate 3-hydroxylase encoded by the *C3H* gene catalyzes the direct 3-hydroxylation of 4-coumarate to caffeate in lignin biosynthesis. In fact, the activity of p-coumarate 3-hydroxylase causes the transition from the pathway of biosynthesis of P-lignin units to the biosynthesis of all other lignin units: C, G, SH, S -lignin monomers [39,40]. *C3H1* gene was expressed both in the tyrosine-supplemented and phenylalanine-supplemented nutrient media.

Despite the fact that the studied genes are divided into two groups (genes involved in lignin biosynthesis and genes involved in flavonoid biosynthesis), these groups are, in fact, overlapping in the “results” column. Phenylalanine ammonia-lyase, 4-coumarate CoA ligase, and cinnamate 4-hydroxylase are key enzymes involved in the “preparation” of amino acid substrates for further involvement in flavonoid biosynthesis [41,42]. It was worth noting that plants transferred to the phenylalanine-supplemented medium enhanced the expression of all selected genes associated with flavonoid biosynthesis. On the contrary, tyrosine did not lead to a significant increase in the number of the following gene transcripts: *CHI*, *F3H*, *DFR*. Such an effect made it possible to assume that tyrosine was less involved in flavonoid biosynthesis, at least in the early stages of wheat ontogenesis. These data correlated well with the total phenols content in experimental plants. Thus, the total phenols content in the plants exposed for 4 h on a medium with 500 μM phenylalanine concentration was almost 2 times higher compared with the plants cultivated on a tyrosine-supplemented solution.

Short-term amino acid exposure (for up to 8 h) was sufficient for the specific phenotypic responses to develop in plants. Thus, exposing plants to Phe and Tyr led to a significant (*p* ≤ 0.05) change in phenylpropanoid level. It was worth noting that the formation of the post-PAL phenolic compounds was “branched”; therefore, both phenylalanine and tyrosine could influence their level, at least indirectly.

The advantages of this approach are obvious for a scaled-up experiment as well [43]. Thus, treating plants with various metabolic intermediates could be a great tool to implement for agriculture. Increased flow through Tyr and Phe could lead to the formation of more resistant plants and contribute to the accumulation of valuable plant products.

## 4. Materials and Methods 

### 4.1. Triticum aestivum L. Cultivation and Experimental Design

Wheat (*Triticum aestivum* L.) “Vánek” variety was obtained from the Seed station at the Ministry of Agriculture of the Kaliningrad region. The seeds were sterilized by incubating for 2 h in 10% (*v*/*v*) NaClO, then washed 10 times with distilled water.

For the laboratory experiments, *Triticum aestivum* L. seeds were sprouted in distilled water. The 3–4-day-old seedlings were transplanted onto perlite impregnated with the 50% Hoagland nutrient solution. Plants were grown under fluorescent lamps at a photon flux density (PFD) of 200 μmol m^−2^ s^−1^, for a 16 h photoperiod, and at a temperature of +25 °C.

In order to study the separate effects of tyrosine and phenylalanine on the metabolic processes of plants, there were used solutions of these substances in concentrations of 100, 200, 300, 400, 500, 600, 800 μM. The 30-day-old plants were transferred to experimental solutions (supplemented with the corresponding amino acids). Wheat plants used in the current experiment reached phases 4 and 5 on the Feekes Growth Stages classification scale. The key features of these phases are the initiation of erect growth, shoot formation, and secondary root system development [44]. Plants were exposed on these substrates supplemented with active substances for 1, 2, 4, and 8 h. As a control, the 50% Hogland solution was used. Plant leaves were used for further experiments.

### 4.2. Reagents

All analytical-grade chemicals used in the assay were obtained from commercial sources. Bi-distilled water was used throughout the experiment. Standard solutions were prepared by dilution of the stock solution.

### 4.3. Tyrosine and Phenylalanine Assay

Tyrosine and phenylalanine were determined by the HPLC method.

Pre-column derivatization.

The pre-column derivatization using *o*-phthalaldehyde was carried out according to the previously developed method [45]. To achieve that, 70 μL of extracts or given standard solutions were mixed with 10 μL of o-phthalaldehyde (OPA) reagent at 25 °C for exactly 120 s, following which the mixture was immediately analyzed by HPLC.

The separation by HPLC was performed according to the previously designed method [46], using a Shim-pack GIST-HP C18 column (internal diameter 150 × 3 mm, particle size 5 μm) (Shimadzu, Kyoto, Japan). Comparing the amino acids retention time and UV spectrum with those of amino acids’ standards (tyrosine and phenylalanine, respectively) confirmed their chromatographic peaks.

### 4.4. Phenolic Compound Assays

Plant extract preparation for phenolic compound assays included homogenization of 0.1–0.2 g of plant sample material with 10 mL of 96% ethanol solution and centrifugation of the mixture at 4500× *g* for 30 min. The supernatant fluid was used for analysis [47].

#### 4.4.1. Total Catechins Content (TCC)

The vanillin method was used to determine the catechin content [48]. First, 4 mL of vanillin reagent was mixed with 1 mL of plant extract in test tubes, using 1 mL ethanol instead of the extract as a blank solution, and subsequently, the tubes’ contents were transferred to the cuvettes. The absorbance measurement was performed after adding the extract to the vanillin reagent. The absorption of the obtained complex between vanillin and plant sample catechins was measured at 520 nm (UV-3600, Shimadzu, Kyoto, Japan). The results were presented as μg of catechin equivalent per g of dry weight (μg CE g^−1^).

#### 4.4.2. Total Proanthocyanidins (PAs) Content 

Proanthocyanidins (PAs) content was measured using butanol–hydrochloric acid assay [49]. First, 0.5 mL of the ethanolic extract was added to 3 mL of butanol–hydrochloric acid reagent (C_4_H_9_OH/HCl, 95:5; *v*/*v*) and 0.1 mL of 2% ferric reagent, after which the test tubes were vortexed and heated in a boiling water bath for 1 h. The absorbances of result mixture were measured at 550 nm (UV-3600, Shimadzu, Kyoto, Japan) against the blank solution. The blank sample contained 0.5 mL of ethanol solvent instead of the extract. The results were presented as μg of cyanidin equivalent per g of dry weight (μg CyE g^−1^).

#### 4.4.3. Total Phenolic Content (TPC)

Total phenolics content was determined by the Folin–Ciocalteu method [50]. Briefly, 100 μL of the extract was mixed with 300 μL of 0.2 M Folin–Ciocalteu reagent and incubated for 10 min under shading conditions. After that, 6 mL of 6.75% sodium carbonate (Na_2_CO_3_) solution was added to each tube, and the tubes were incubated for 30 min, also in shading conditions. The absorbance of the resultant mixture was measured at 765 nm (UV-3600, Shimadzu, Kyoto, Japan). TPC was presented as μg gallic acid equivalent per gram dry weight (μg GAE g^−1^).

In order to reduce the number of experimental variants when evaluating the enzymatic activity and gene expression involved in lignin biosynthesis, there were chosen amino acid concentrations and exposure times that best affected phenolic compounds accumulation and also significantly affected the total pool of free tyrosine and phenylalanine in the experimental plants.

### 4.5. Assays of Enzyme Activities

#### 4.5.1. PAL and TAL activity assays

Protein extractions and kinetic assays were carried out according to the methods described by Cheng and Breen [51] and Rösler et al. [52] with some modifications. Plant material (0.2 g) was stored at −80 °C after flash-freezing by exposure to liquid nitrogen. Frozen plant material was ground and treated with 1 mL of acetone, then incubated at −20 °C for 15 min. Next, the mixture was centrifuged at 16,000× *g* (15 min, 4 °C). The pellets were obtained by low-speed rotation at 4 °C in the presence of 100 mM sodium borate pH 8.8/2 mM EDTA solution. After 60 min, another centrifugation was carried out, and the supernatant was used for kinetic analyses.

Substrates—l-phenylalanine and l-tyrosine—and products—trans-cinnamic acid (TCA) and p-coumaric acid (PCA)—were used as standards, respectively.

PAL activity was quantified by the production of TCA, which was controlled by taking absorption at 290 nm [52] every minute up to 20 min at 37 °C (UV-3600, Shimadzu, Kyoto, Japan). The mixture contained 61 mM l-phenylalanine, 30 mM sodium borate buffer (pH 8.8), and 75 μL of plant extract, making the total volume of the resultant mixture as 1 mL. L-phenylalanine (substrate) was added after a 10 min pre-incubation at 37 °C. Plant extract previously incubated in a buffer without substrate was used as the blank solution.

TAL activity was quantified in a similar manner by monitoring the rise of the PCA level at 310 nm [52]. The 1 mL assay mixture consisted of 1.9 mM l-tyrosine, 30 mM sodium borate buffer (pH 8.8), and 75 μL of plant extract. All other conditions and procedures coincided with the protocol of the PAL assay. Enzyme activity was expressed as unit per mg of protein. One unit of PAL activity was determined as the amount of the enzyme that produced 1 nmol TCA per h. One unit of TAL activity was determined as the amount of the enzyme that produced 1 nmol PCA per h.

#### 4.5.2. Peroxidase Activity Assay

Assay of peroxidase was performed using the Malik and Sing method with some modifications [53]. First, 100 μL of extract and 1 mL of *o*-dianisidine solution were added to 2 mL of phosphate buffer (pH 6/pH 7). The reaction was started by adding 100 μL of 0.2 mM hydrogen peroxide (H_2_O_2_), and subsequently, the absorbance was recorded at 460 nm every 30 s for up to 5 min. The enzyme activity was performed using an extinction coefficient of *o*-dianisidine. The POD activity was expressed as unit per mg of protein. Protein concentrations for both tests were determined by the Bradford method [54].

### 4.6. Gene Expression 

RNA Extraction, RT-PCR, and qRT-PCR

Total RNA of the plant samples of wheat cultivars were extracted using TRIzol (“Invitrogen”, Waltham, Massachusetts, USA), as described before [55]. For cDNA synthesis, RNA samples were treated with DNase 1 (Thermo Scientific, Waltham, MA, USA). The 10 μL reaction mixture containing 1 μg of RNA, 1 μL 10x of reaction buffer, 1 e.a. of DNase was incubated at 37 °C for 15 min. DNase was inactivated by the addition of 2.5 mM of EDTA and heating at 65 °C for 10 min. Synthesis of single-stranded cDNA from total RNA was performed using the RevertAid H Minus First Strand cDNA Synthesis Kit (Thermo Scientific, USA) according to the manufacturer’s guidelines. Spectrophotometric measurement of cDNA concentration was carried out using the spectrophotometer.

Real-time polymerase chain reaction (PCR) was performed on the CFX96™ Real-Time System (Bio-Rad, USA) using an intercalating dye SYBRGreen I (“Invitrogen”, Waltham, MA, USA). Primer sets were designed from wheat sequences deposited in GenBank (Table 1) [56]. The ΔΔCt method was used to estimate the relative expression levels of the analyzed genes [57].

It has been shown that the expression of *PAL6*, *C4H1*, *4CL1*, *C3H1,* in particular, has a significant correlation with the lignin content [37], and *CHS*, *CHI*, *F3H*, and *DFR* genes reliably correlate with the level of phenolic compounds of wheat plants [58]; for this reason, these genes were selected for analysis. The *ARF* (ADP-ribosylation factor) gene and actin were used as the reference gene.

### 4.7. Statistical analysis

The values are means of at least three replicates. The level of significance was established at a *p*-value of *p* ≤ 0.05. Data were statistically analyzed by using the SigmaPlot 12.3 (Systat Software GmbH, Erkrath, Germany). The Shapiro–Wilks test was used for normality checking; additionally, the experimental data were checked for the homogeneity of variance. These tests allowed us to switch on one-way ANOVA, which was presented for each factor separately (exposure time, amino acid concentration). The results were presented as mean ± standard deviation (*n* = 3). The graphs were prepared using OriginPro 9 (OriginLab Corporation, Northampton, MA, USA).

## 5. Conclusions

The current study analyzed the effect of aromatic proteinogenic amino acids (phenylalanine and tyrosine) on the secondary metabolism of *Triticum aestivum* L. The involvement of amino acids through the PAL-associated metabolic pathways, such as biosynthesis of flavonoids and the formation of lignin, led to increased production of secondary compounds of a phenolic nature. Such changes could be considered from two perspectives; on the one hand, secondary compounds are characteristic inducers of plant resistance, and, on the other hand, intensification of the flux through the key enzyme of the phenylpropanoid pathway allows the accumulation of physiologically active substances necessary for human consumption. Thus, the use of phenylalanine and tyrosine as an element of plant treatment could be an excellent agricultural tool.

## Figures and Tables

**Table 1:** Lists of genes and primers for qRT-PCR amplification.

**Lignin-Related Genes**		
	**Primer**	Sequence (5′-3′)
***PAL6***	*PAL6-F*	CTCAAGCTCATGTCCTCCACA
	*PAL6-R*	TCAGCACCTTCTTCGACACC
***C3H1***	*C3H1-F*	GGCTGTGTCCACTTAATG
	*C3H1-R*	TGTCATCACTAAGGTCATAC
***C4H1***	*C4H1-F*	CAGCCTCCACATCCTCAAG
	*C4H1-R*	CTTAGGACGAGCGAACAATC
***4CL1***	*4CL1-F*	CACTCAGCCAGCCAGCAG
	*4CL1-R*	ACATTACACAAGCAGGAAGAACC
**Phenolic Compound-Related Genes**		
***CHS***	*CHS-F*	CTCATGATGTATCAGCAGG
	*CHS-R*	ACATCCTTGAGGTGGAA
***CHI***	*CHI-F*	GCAGTACTCGGACAAGGTGA
	*CHI-R*	GTTCGTTCACACCGAAACC
***F3H***	*F3H-F*	CCTACTTCTCGTACCCGGTG
	*F3H-R*	GAACGTCGCGATCGACAG
***DFR***	*DFR-F*	TGCTGGAGCTTCCCGGAGC
	*DFR-R*	CGTGGGGATGATGCTGATGA
**Reference Genes**		
***Actin***	*ACT-F*	GCCACACTGTTCCAATCTATGA
	*ACT-R*	TGATGGAATTGTATGTCGCTTC
***ARF***	*ARF-F*	CTGACGCCGAGGATATCCA
	*ARF-R*	GCCTTGACCATACCAGTTCCA

## Supplementary Materials

The following are available online at https://www.mdpi.com/2223-7747/9/4/476/s1, Figure S1: Partial alignment of amino acid sequences for given PAL and PTAL proteins (BdPAL1, TaPAL6, TaPAL1).

## Abbreviations

4CL—4-coumarate CoA ligase;
ARF—ADP-ribosylation factor;
C3H—p-coumaroyl shikimate/quinate 3′-hydroxylase;
C4H—cinnamate 4-hydroxylase;
CHI—chalcone isomerase;
CHS—chalcone synthase;
DFR—dihydroflavonol 4-reductase;
F3H—Flavanone 3-hydroxylase;
PAL—phenylalanine ammonia lyase;
PCA—p-coumaric acid;
POD—peroxidase;
qRT-PCR—quantitative real-time PCR;
TAL—tyrosine ammonia-lyase;
TCA—trans-cinnamic acid.
