# Supplementary material for: Phenylalanine and Tyrosine as Exogenous Precursors of Wheat (Triticum aestivum L.) Secondary Metabolism through PAL-Associated Pathways
Source: Plants (Basel). 2020 Apr 9;9(4):476. doi: 10.3390/plants9040476 (PMC7238280; doi:10.3390/plants9040476)
Supplement: Supplementary file 1 [file plants-09-00476-s001.pdf]

Supplement figures

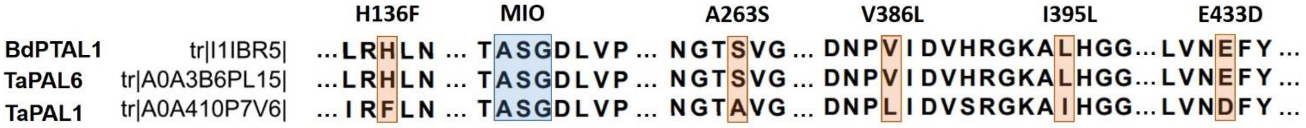

Figure S1. Partial alignment of amino acid sequences for given PAL and PTAL proteins (BdPAL1, TaPAL6, TaPAL1). Amino acids (ASG) turned into the MIO cofactor, and groups supposedly defining the substrate selectivity between TAL and PAL (H136F, A263S, V386L, I395L, D433E) are highlighted with blue and brown respectively
